# The Survival Rate of Posterior Immediate Implants in the Maxilla and Mandible: An Observational Retrospective Study of 158 Dental Implants

**DOI:** 10.7759/cureus.45579

**Published:** 2023-09-19

**Authors:** Lanka Mahesh, Ana Boquete Castro, Meenu T Bhasin

**Affiliations:** 1 Implantology, The Specialist Clinic, Delhi, IND; 2 Periodontics, Miguel de Cervantes European University, Valladolid, ESP; 3 Periodontics, Dr. Bhasin's Super Speciality Dental Clinic and Implant Centre, Delhi, IND; 4 Periodontics, Sudha Rustagi College of Dental Sciences and Research, Delhi, IND

**Keywords:** implants, immediate placement, retrospective analysis, extraction socket, implant survival

## Abstract

Background: Immediate implants are popular for the anterior sextants of the mouth and have shown a high success rate for the same. However, their installation in a fresh extraction socket in the posterior segments can also be beneficial to the patients and limit the time for the patient to start the masticatory function. However, there have been contradicting results in different studies.

Objectives: The primary objective of this retrospective study was to establish correlations between factors such as implant dimensions, implant categories, implant location, and various demographic parameters in relation to the longevity of implants. This investigation was conducted through a comprehensive clinical evaluation of immediate implants situated within the molar sections of both the upper (maxillary) and lower (mandibular) jaws.

Methods: Between October 2015 and August 2022, a total of 158 implants were implanted, with 87 males and 71 females undergoing implant placement following tooth extraction. All implants were reinstated between 12 and 18 weeks after they were placed. Inferential statistics were performed using SPSS Statistics version 23 (IBM Corp. Released 2015. IBM SPSS Statistics for Windows, Version 23.0. Armonk, NY: IBM Corp.). The Chi-square test was employed to determine statistical significance (p=0.05) between survived and failed implants in relation to various study factors. Lastly, in order to measure the survival rate under different time periods after implant placement, the life table method and Kaplan-Meier survival rate analysis were used.

Results: Success of implants was observed at 149 sites, whereas implant failure was seen at nine sites in total. From placement to loading, the implant failed at five sites, and the cumulative survival rate was found to be 96.83%, from loading to one year, implant failure was seen at three sites, and the cumulative survival rate was found to be 94.9%, from one to two years after loading implant failure was seen at only one site with cumulative survival rate to be 94.93%. From two to three years after loading, implant failure was not seen at any site.

Conclusions: Regardless of implant size or insertion location, rapid implant implantation in fresh extraction sockets can result in predictable clinical outcomes.

## Introduction

Currently, Implant placement is a well-known choice to replace missing teeth. Since the time Branemark introduced dental implants in 1977, a staged protocol had been well documented and followed with, implant placement in a healed extraction socket with delayed implant loading [1]. However, patience was required on the patient’s part, due to a longer waiting period from tooth extraction to the placement of the implant in the healed alveolar ridge and later for osseointegration and loading required [2]. Over the periods, there has been a paradigm shift in the treatment protocols, and immediate implants are now a well-accepted norm. According to the “Glossary of Implant Dentistry of the International College of Oral Implantologists,” immediate implant placement is defined as “placement of a dental implant at the time of tooth extraction, into the extraction socket” [3]. A recent systematic review by Garcia-Sanchez et al. reported no difference in the cumulative similar survival rate between immediate and delayed implants into healed extraction sockets [4].

In the 2018 consensus meeting of ITI held in Amsterdam, the ITI statement mentions a 98% survival rate of immediate implants with immediate or early loading and about 96% (median 99%, range 91-100%) with immediate implants and conventional loading [5]. Immediate implant placement offers several advantages over delayed protocol, including fewer patient visits due to a decreased number of surgical procedures leading to a reduction in the treatment charges and decreased treatment time required, which has provided a positive psychological impact on the patient [6-8]. The ability to place the implant in an ideal axial position concerning the tooth is another benefit [7]. However, there are many challenges that a clinician encounters in immediate implant placement post-extraction, which include increased complication during surgery, insufficient quality and quantity of bone and soft tissues, resulting in poor aesthetic outcomes, difficulty in achieving primary stability, implant angulation challenges, an increased risk of infection, and conflicting findings related to long term survival of implants installed by this approach [4,9,10].

The success of immediate implants is commonly defined by implant survival. Whenever an implant shows clear signs and symptoms that require its removal, it is considered a failed implant. This can happen either before loading or after loading the implant [11].

Dental implants are prosthetic tooth roots that are surgically implanted into the jawbone to support replacement teeth. The term posterior signifies that the research focuses on implants placed at the back of the mouth. The term immediate implants refers to implants that were inserted soon after tooth extraction, which can decrease the need for several surgical procedures. The study evaluates implants in two separate locations: the maxilla (upper jaw) and the mandible (lower jaw). These areas may have different anatomical and functional distinctions that may influence implant placement.

The term observational retrospective study refers to a study that analyses data obtained from previous cases without the intervention or modification of the researchers. The term "observational" implies that the researchers are watching current situations rather than actively changing variables. The term retrospective refers to a study that looks back at previous data. A total of 158 dental implants were used in the investigation. The number of individual implants being studied for their survival rate is indicated by this sample size.

Overall, the title suggests that the study intends to investigate how well dental implants implanted shortly after tooth extraction in the back portions of the upper and lower jaws have performed over time. The findings could provide useful insights into the success of this particular strategy for implant placement and assist in educating clinical practices related to dental implant treatments in the posterior regions.

## Materials and methods

All patients who received dental implant placement during seven years, spanning from October 2015 to August 2022, at a private dental clinic, were included in the current retrospective analysis.

The information was taken from the patient’s electronic dental records at the facility. Inclusion criteria for the study were immediate implant placement on sites, where the teeth were deemed non-restorable due to decay, untreatable endodontic lesions, or a vertical fracture, as well as maxillary molar and mandibular molar sites for implant insertion and implants that had primary stability measured with a manual torque wrench >=30NCm. The exclusion criteria for the study were medically compromised patients (ASA categories 3 and 4), patients receiving head and neck radiation therapy, those with a history of drug abuse or prolonged steroid use, those with marked local pathology at the surgical site, marked local pathology at the site of surgery, or severe periodontal destruction, or cases requiring grafting where the walls were missing.

A comprehensive total of 158 dental implants were meticulously positioned, with 87 implants in male participants and 71 implants in female participants. These implants encompassed a range of tapered dimensions in terms of length and diameter and belonged to established brands such as Nobel Biocare (NobelActive) and Bioner (Bioner TOP DM). To discern any anatomical factors that might pose risks and to evaluate the feasibility of immediate implant placement, an exhaustive clinical assessment was conducted, accompanied by the creation of diagnostic models. Furthermore, preoperative radiographs and/or cone-beam computed tomography scans were meticulously acquired for each participant.

All implants introduced within the dental clinic adhered to the bone-level implant design. To optimize the preservation of the buccal plate, a crucial consideration, atraumatic extractions were executed, and mucoperiosteal flap elevation was deliberately avoided. Precautions were taken to safeguard both the interradicular and buccal bone during the extraction process, wherein the tooth was partitioned into manageable segments to enable the individual removal of roots with minimal disturbance. Following extraction, the socket was probed to ensure the integrity of the outer walls, free from dehiscence defects. Subsequently, the interradicular bone was meticulously prepared in accordance with the manufacturer's prescribed guidelines for implant placement.

All implants were positioned 1.5 mm below the bone crest, followed by the placement of a healing abutment. The surgical site was sutured using 4.0 Vicryl sutures, with the flap not advanced to ensure optimal healing conditions. Comprehensive post-operative instructions were communicated to patients, accompanied by a prescription of amoxicillin 500 mg thrice daily and paracetamol 500 mg for a week to manage potential discomfort. Additionally, a prescription of chlorhexidine 0.12% mouthwash was provided, with patients advised to rinse twice daily for 15 days. A follow-up appointment was scheduled for two weeks post-implant placement to remove sutures and assess the progress of the healing process.

After 12 weeks following the implant placement, a conclusive impression was taken using a personalized impression tray and a pick-up impression coping. This impression, capturing the exact positioning of the implants, was subsequently forwarded to a dental laboratory to facilitate the crafting of the crown components. Notably, all permanent prosthetic restorations were designed to be secured using screws, ensuring robust attachment, and these restorations were tightened to a torque of 30 Ncm utilizing a meticulously calibrated torque driver.

All patients had annual follow-ups following implant implantation. Patients were clinically assessed for implant stability and peri-implant inflammation assessing the signs of inflammation and any increase in depths using a plastic periodontal probe at each subsequent appointment. Radiographs of the implant site were taken at each follow-up visit. A record was made of the patient’s date of surgery, gender, age at the time of surgery, site of placement, type of implant, number of implants, length, and width of implants, date of prosthesis placement, and date of implant loss.

## Results

Results of the present study showed that there was a total of 158 implants placed. Comparatively, implants were placed more among males and among the 20-30-year-old age group in mandible second molars. The majority of the implants that were placed had a diameter of 5 mm and a length of 10 mm and were single individual implants and placed immediately after extraction. For the majority of the subjects, the loading period was found to be 13-26 months (Table 1).

**Table 1:** Descriptive data

		Frequency	Percent
Gender	Female	71	44.9
	Male	87	55.1
Age group	20-30	24	15.2
	31-40	23	14.6
	41-50	23	14.6
	51-60	23	14.6
	61-70	20	12.7
	71-80	22	13.9
	>80	23	14.6
Mandible	First molar	28	36.8
	Second molar	48	63.2
Maxilla	First molar	45	54.9
	Second molar	37	45.1
Diameter	4.0	60	38.0
	4.3	35	22.2
	5.0	63	39.9
Length	10.0	123	77.8
	11.5	35	22.2
Type of implant	Individual	95	60.1
	Splinted	63	39.9
Placement time	Delayed	63	39.9
	Immediately	95	60.1
Prosthetic type	Multiple	63	39.9
	Single	95	60.1
Duration in months	0-12	32	20.3
	13-24	35	22.2
	25-36	63	39.9
	37-42	28	17.7
Loading period in months	0-12	32	20.3
	13-24	63	39.9
	25-36	63	39.9

**Figure 1:**
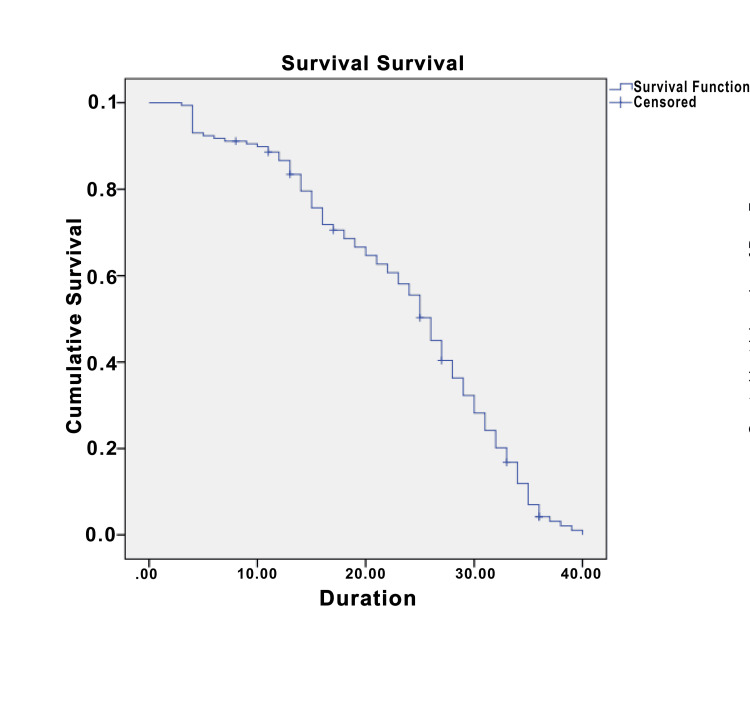
Kaplan-Meier survival analysis

The success of implants was observed at 149 sites, whereas implant failure was seen at nine sites in total. From placement to loading, the implant failed at five sites, and the cumulative survival rate was found to be 96.83%, from loading to one year, implant failure was seen at three sites, and the cumulative survival rate was found to be 94.9%, from one to two years after loading implant failure was seen at only one site with the cumulative survival rate to be 94.93%. From two to three years after loading, implant failure was not seen at any site (Tables 2-3, Figure 1).

**Table 2:** Kaplan-Meier survival analysis

Total N	N of events (survived)	Censored	
		N	Percent
158	149	9	5.7%

**Table 3:** Life table method

Loading time	Number entering interval	Number withdrawing during Interval	Survived implant	Survival rate in the interval	Cumulative proportion surviving at end of interval (%)
Placement to loading	158	5	153	96.83	96.83
Loading to 1 year	153	3	150	98.0	94.9
Loading 1-2 year	150	1	149	99.3	94.93
Loading 2-3 year	149	0	149	100	94.30

No significant difference was seen in the rate of implant failure or survival among different age groups, gender, arches, sites, diameter and length of implant, type of implant, and time of implant placement (Table 4).

**Table 4:** Survival rate of implants according to the variables

	Failed (%)	Survived (%)	p-value
Female	5.6	94.4	0.626
Male	5.7	94.3	
20-30 years old	0	100	0.170
31-40 years old	13	87	
41-50 years old	4.3	95.7	
51-60 years old	8.1	91.3	
61-70 years old	0	100	
71-80 years old	0	100	
>80	13	87	
Mandible	6.6	93.4	0.452
Maxilla	4.9	95.1	
First molar	6.8	93.2	0.405
Second molar	4.7	95.3	
Diameter 4	5	95	0.949
Diameter 4.3	5.7	94.3	
Diameter 5	6.3	93.7	
Length 10	5.7	94.3	0.633
Length 11.5	5.7	94.3	
Individual implant	6.3	93.7	0.484
Splinted implant	4.8	95.2	
Delayed placement	4.8	95.2	0.484
Immediate placement	6.3	93.7	
Multiple implants	4.8	95.2	
Single implant	6.3	93.7	0.484

## Discussion

Historically, greater attention has been given to immediate implants in the aesthetic zone, with many scholars demonstrating accelerated approaches with improved outcomes [12-15]. This is in contrast with the paucity of evidence of implant survival about immediate implants in the posterior sextant, which could be because immediate implants in the posterior region are quite challenging due to the presence of vital anatomic landmarks. Difficulty in achieving primary stability is another factor the clinicians are hesitant to do immediate implant placement in this area.

However, over time, various techniques have been proposed to overcome the challenges of immediate molar replacements, and they are currently regarded as a predictable treatment strategy, with survival rates similar to implants placed in healed ridges [7,16].

In their 11-year retrospective study of 300 immediately placed implants in molar extraction sockets, Schwartz et al. [17] reported a 97.3% overall survival rate. They credited the success to meticulous surgery, the preservation of the molar socket architecture, less traumatic extraction procedures, and precise osteotomy preparation to obtain the proper implant orientation and primary stability. Fugazzotto et al. [18] reported a survival rate of 99.5% in their study on the replacement of single maxillary molars.

Atieh et al., in another systematic review on the same, estimated survival rates of 99% with immediate implants. The maximum site where the implant was placed was molars (65.2%), and no grafting was done with implant placement [10].

The present study is retrospective, in contrast to the abovementioned prospective study wherein there is a possibility of an inherent bias on the part of the investigator and operator. In the present retrospective study, the patients were followed for five years. Fewer patients missed the in-between appointments. The patients who missed their follow-ups were traced, contacted, and scheduled for clinical examination at the end of their five-year follow-up. The success rate was 98.1%, with three cases of implant failure (98% for the maxilla and 99% for the mandible). In the current investigation, two maxillary implants failed before implant loading, although the cause could not be isolated to any one particular reason. One failure was detected in the fourth year, and the possible reason could be attributed to excessive load as the patient had missing teeth on the contralateral side either the same arch or opposite, and had unilateral chewing, causing excessive loading on the single implant. Smith et al. [19] in their 11-year retrospective analysis also reported a high percentage of success, like the present study.

Our study is in contrast with reports from Meijer et al. who reported high implant failure. It can be assumed that a lack of primary stability along with impaired healing and decreased initial contact between implant and bone are the main reasons for the failures [20]. Quiryen et al. also reported a 4-5% total incidence of implant loss after immediate placement, with a greater incidence when a combination of immediate placement and loading was performed, especially for minimally rough implant surfaces [21].

The key factor influencing implant survival is peri-implant bone resorption. During the follow-up period, the presence of peri-implant disease was assessed for each implant included in this retrospective analysis.

The high success rate in the present study could be attributed to meticulous planning, flapless approach, maintenance of inter radicular bone, presence of intact outer bony walls, adequate implant stability, and regular follow-ups.

However, there are certain limitations of the study, which have been linked to the retrospective study design, reliability of secondary data, and random case selection. In addition, the study didn’t include the effect of other compounding factors like cases requiring grafting.

## Conclusions

The long-term survival rate of the immediately placed implant is excellent. Immediate implant placement reduces the trauma associated with surgery for patients by placing implants at the same time as tooth extraction. The total treatment duration is shortened by half. Immediate implant placement can generate a pleasing prosthesis by conserving as much of the extraction socket as possible.
